# Posterior reversible encephalopathy syndrome (PRES) caused by chemotherapy containing S-1 against diffuse type gastric cancer

**DOI:** 10.1007/s12328-020-01254-w

**Published:** 2020-10-03

**Authors:** Masashi Yokota, Takayuki Shirai, Masaya Sano, Hiroyuki Ito, Junko Nagata, Hitoshi Ichikawa, Seiichirou Kojima, Shinji Takashimizu, Norihito Watanabe

**Affiliations:** Department of Gastroenterology, Hachioji Hospital, Tokai University School of Medicine, Tokyo, Japan

**Keywords:** PRES, Chemotherapy, S-1

## Abstract

A 23-year-old woman who complained of abdominal distension and anorexia was referred to our hospital. Computed tomography showed ascites, a huge hepatic tumor and ovarian tumor. Gastroscopy revealed type 4 gastric cancer and biopsy examination showed poorly differentiated adenocarcinoma with signet ring cell carcinoma. We diagnosed her with stage IV advanced gastric adenocarcinoma. She received the chemotherapy with S-1 and CDDP regimen. After two courses, this regimen was changed to the SOX (S-1 + OHP) regimen because of acute kidney injury. After one course of the SOX regimen, she developed general muscle cramp. Magnetic resonance imaging showed a 15 mm, round, high-intensity signal at the parietal lobe on T2-weighted images. She was hospitalized for with the suspicion of brain metastasis. Anticonvulsants improved her muscle cramp, but she had consciousness disturbance on the 9th hospital day. T2WI showed high-intensity signals within the cerebral white matter at both sides of the occipital lobe. We suspected leukoencephalopathy caused by S-1 and discontinued the SOX regimen. We also treated her hypertension and hyponatremia. Her consciousness disturbance improved in several days, and the T2WI finding was markedly improved on the 20th hospital day. We diagnosed her with posterior reversible encephalopathy syndrome caused by chemotherapy containing S-1.

## Introduction

Patients with posterior reversible encephalopathy syndrome (PRES) have several symptoms such as headache, cramp, consciousness disturbance, and visual disturbance due to increasing vascular permeability and vascular endothelial cell disorder caused by intra cerebral hypertension. PRES is caused by several factors including chemotherapy. Meanwhile, S-1 is the key drug for the treatment of advanced gastric cancer.

The development of PRES is related to the length of administration and the dose of drugs. However, in the present case, the dose of the drug was not high and the period of administration was not long.

## Case report

**Fig. 1:**
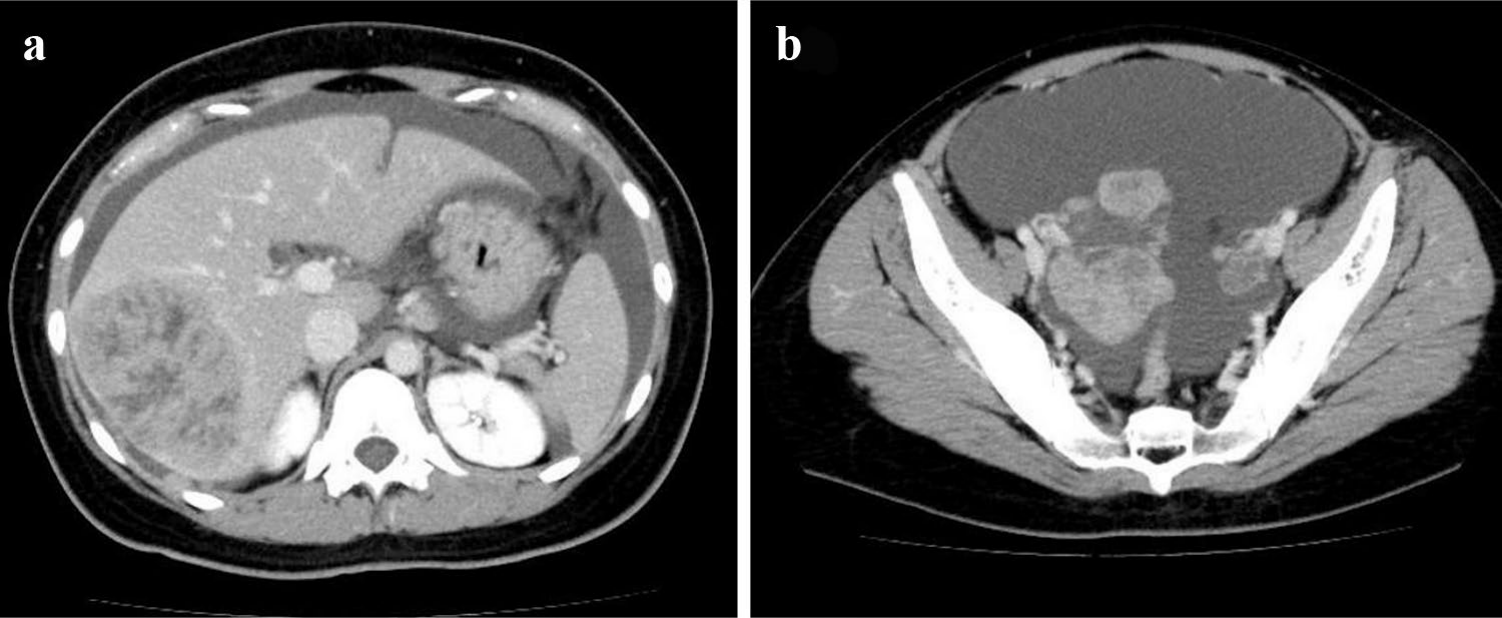
Abdominal CT at presentation at our hospital. **a** Abdominal CT revealed huge hepatic tumor in the right lobe. **b** A moderate amount of ascites, and a right ovarian tumor was also seen

**Fig. 2:**
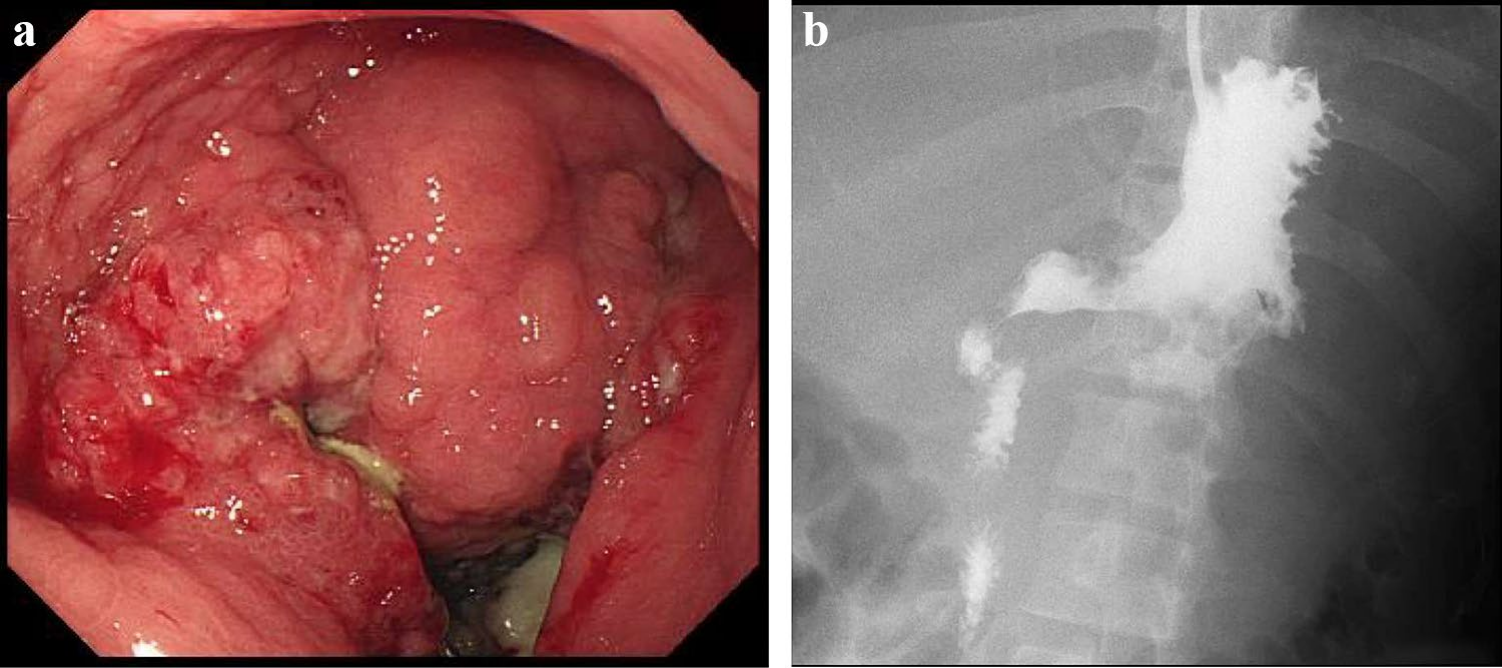
**a** Gastroscopy showed an irregular-shaped erosion, ulceration, and giant folds in the gastric body. **b** Upper gastrointestinal (GI) series showed poor distensibility of gastric wall in the antrum and the body of the stomach

**Fig. 3:**
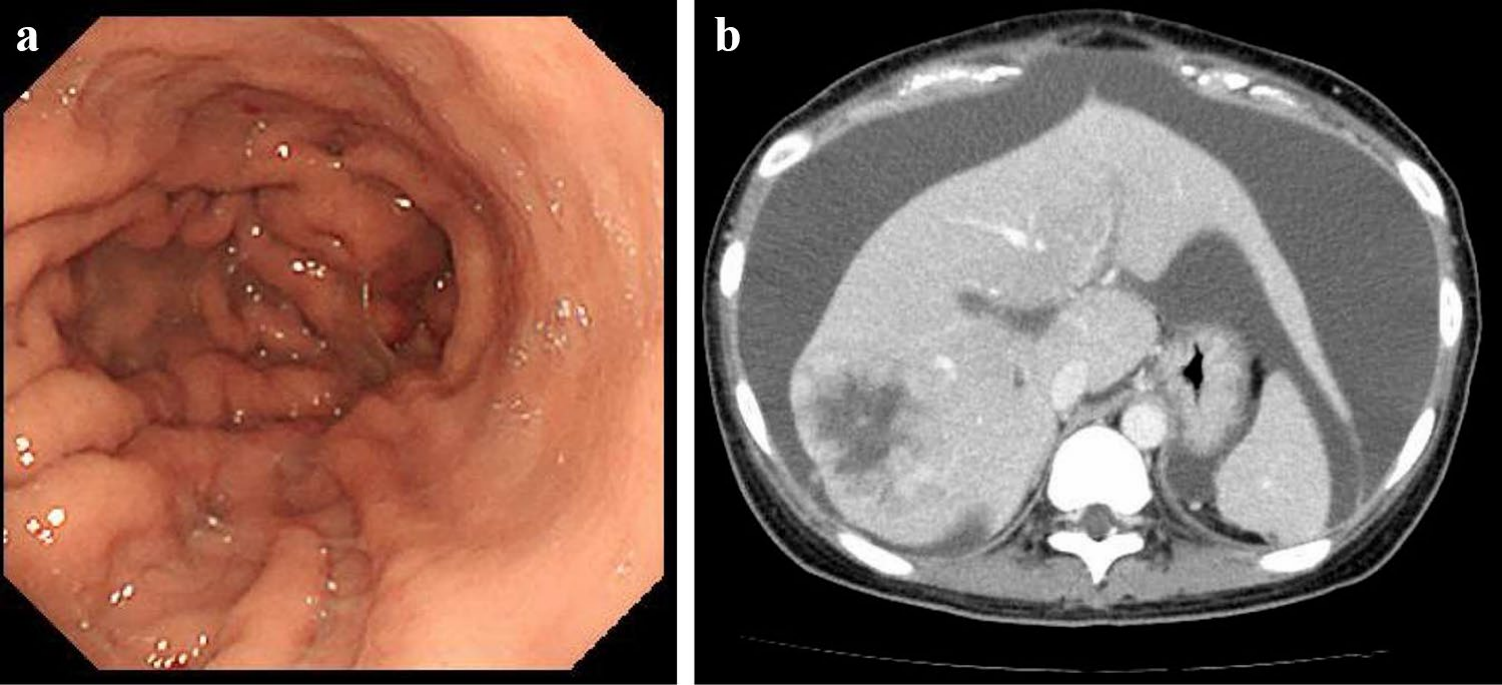
Gastroscopy and abdominal CT after chemotherapy. **a** After the chemotherapy, mucosal swelling, and thick folds were diminished and ulceration was reduced in size on gastroscopy. **b** The hepatic tumor decreased in size on the CT

**Fig. 4:**
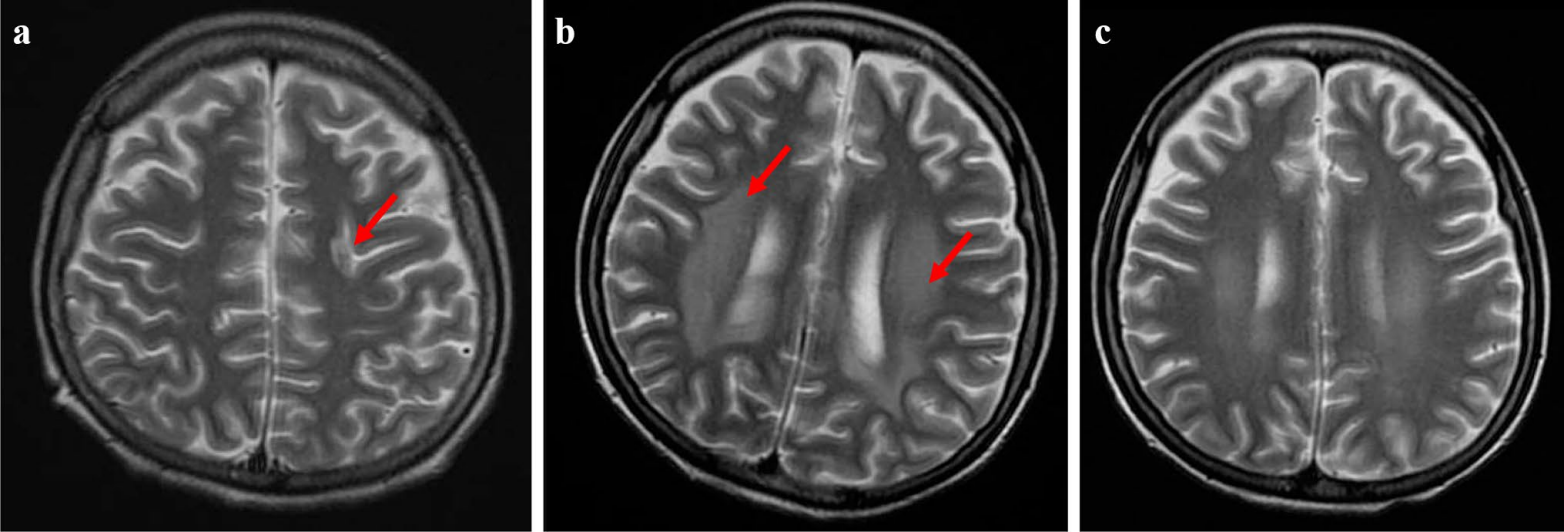
T2-weighted images on head MRI. **a** A round, high-intensity signal (arrow) was recognized in the white matter in the left parieto-occipital area on the 78th day after the start of chemotherapy and she was admitted to our hospital. **b** High-intensity signals (arrows) within the cerebral white matter are observed at both sides of the occipital lobe on the 9th hospital day. **c** After the chemotherapy, this lesion gradually disappeared. This MRI image was obtained on the 20th hospital day

A 23-year-old woman visited a local medical doctor with abdominal distension and anorexia. Computed tomography (CT) showed ascites, a huge hepatic tumor, and an ovarian tumor (Fig. 1a, b). Therefore, she was referred to our hospital. The patient had no history of previous illness and no family history of hereditary diseases. She had no habits of smoking and drinking. On physical examination, she looked pale, her palpebral conjunctivae were anemic, and her abdomen was distended. She had anemia with a hemoglobin level of 4.3 g/dl on blood examination, but the levels of tumor markers, CEA, and CA19-9 were normal. CT showed marked gastric mucosal hypertrophy, a large amount of ascites, a huge hepatic tumor and an ovarian tumor. Gastroscopy revealed poor distension of the gastric wall with a large, irregular-shaped ulcer in the gastric body (Fig. 2a), and she was diagnosed with type 4 gastric cancer. Upper gastrointestinal series showed narrowing of the gastric lumen, but contrast agent could pass through the strictured segment (Fig. 2b). Pathological examination of a biopsy specimen revealed poorly differentiated adenocarcinoma with signet ring cell carcinoma (HER2-negative). The final diagnosis was advanced gastric adenocarcinoma, clinical stage IV. She received chemotherapy with a combination regimen of S-1 and CDDP. She developed acute kidney injury; therefore, this regimen was changed to two courses of the SOX (S-1 + OHP) regimen. After she received one course of the SOX regimen, she developed general muscle cramp. Gastroscopy revealed that the mucosal findings were improved and CT showed that the hepatic tumor was reduced in size (Fig. 3a, b). Seventy-eight days after the start of chemotherapy, she was transported to our hospital by ambulance for convulsion. Magnetic resonance imaging (MRI) showed a 15 mm, round signal at the parietal lobe in T2-weighted images (Fig. 4a). She was hospitalized due to suspicion of brain metastasis. Anticonvulsants improved her general muscle cramp, but she had consciousness disturbance on the 9th hospital day. Hypertension (BP: 155/114 mmHg) and hyponatremia (Na: 128 mEq/L in the blood test) were also found. T2-weighted images showed high-intensity signals within the cerebral white matter at both sides of the occipital lobe (Fig. 4b). We suspected leukoencephalopathy caused by S-1; therefore, we discontinued the SOX regimen. Furthermore, we administered an antihypertensive agent and electrolyte replenisher for the treatment of hypertension and hyponatremia. Her consciousness disturbance improved in a few days, and follow-up MRI (T2WI) findings were markedly improved on the 20th hospital day (Fig. 4c). We diagnosed the patient as having PRES caused by chemotherapy containing S-1.

## Discussion

PRES often refers to leukoencephalopathy in which the findings of image diagnosis and clinical course are reversible. It is caused by cerebrovascular edema because of increasing vascular permeability and vascular endothelial cell damage due to hypertension. Patients with PRES have several symptoms such as headache, cramp, consciousness disturbance, and visual disturbance. PRES can be caused by immunosuppressive agents, chemotherapy, eclampsia, collagen disease and so on [1, 2]. A typical finding of PRES on T2-weighted images is a high-intensity signal within the cerebral white matter at the watershed area of the parieto-occipital lobe. However, regions with high intensity on diffusion-weighted imaging (DWI) and low intensity on apparent diffusion coefficient (ADC) map are reported to be irreversible, reflecting cytotoxic edema [3, 4]. The prognosis of PRES is relatively good, and symptoms are improved by treating hypertension and discontinuation of the suspected drug. Many drugs with side effects can cause PRES, especially fluorinated pyrimidines like 5-FU, carmofur, and tegafur. Leukoencephalopathy caused by carmofur was the first reported at the domestic meeting of the Japanese Society of Psychiatry and Neurology in 1982. According to the research conducted by the Ministry of Health, Labor and Welfare, the incidence of PRES was about 0.026% between 1982 and 1995. When we performed a literature search in the database of the Japan Medical Abstracts Society using the keywords of leukoencephalopathy and S-1, only the report of Toda et al. [5] was found. On the other hand, when we performed a literature search in the Pharmaceuticals and Medical Devices Agency (PMDA), there were 58 reports on leukoencephalopathy caused by S-1 between 2004 and 2017. We examined these reports for age, gender, length of illness, and S-1 dose. The outcome was as follows: mean age of the patients was 61.2 years (range, 30–80 years), ratio of males to females was 1–0.9, mean day of onset after starting to take S-1 was 133.6 days (1–787 days), and mean S-1 dose was 47.8 mg/day (30–60 mg/day). Some cases of leukoencephalopathy were reversible, while other cases were irreversible. Deaths have also been reported, and the prognosis varied. According to the research conducted by the Ministry of Health, Labor, and Welfare, in case of carmofur, it was reported that the prognosis of patients who stopped taking the suspected drug earlier and who took a lower amount of the drug per day was relatively good. S-1 is used in many regimens, but it is not well known that leukoencephalopathy is among the side effects of S-1 [6]. The content of tegafur is 300–600 mg/day in UFT, while it is 80–120 mg/day in S-1, which is probably the reason why there are a small number of cases of leukoencephalopathy among patients who take S-1. On the other hand, when searching with PubMed using L-OHP and PRES as keywords, 14 documents were hit. Although in a few cases L-OHP were used alone, the causal relationship was unclear in most cases due to combination therapy including L-OHP. Therefore, we considered that it was caused by S-1 in the present case, because there was an obvious difference of frequency between fluorinated pyrimidines and L-OHP, although we could not completely deny the possibility caused by L-OHP. As the prognosis of PRES depends on appropriate treatment, it is important to diagnose this disease early and reduce or discontinue the suspected drug in order to prevent the development of irreversible disorders.
